# Urinary Signatures of Renal Cell Carcinoma Investigated by Peptidomic Approaches

**DOI:** 10.1371/journal.pone.0106684

**Published:** 2014-09-09

**Authors:** Clizia Chinello, Marta Cazzaniga, Gabriele De Sio, Andrew James Smith, Erica Gianazza, Angelica Grasso, Francesco Rocco, Stefano Signorini, Marco Grasso, Silvano Bosari, Italo Zoppis, Mohammed Dakna, Yuri E. M. van der Burgt, Giancarlo Mauri, Fulvio Magni

**Affiliations:** 1 Department of Health Science, University of Milano-Bicocca, Monza, Italy; 2 Department of Specialistic Surgical Sciences, Urology unit, Ospedale Maggiore Policlinico Foundation, Milano, Italy; 3 Department of Laboratory Medicine, Hospital of Desio, Desio, Italy; 4 Department of Surgical Pathology, Cytology, Medical Genetics and Nephropathology, Azienda Ospedaliera San Gerardo, Monza, Italy; 5 Department of Medicine, Surgery and Dental Sciences, Pathology Unit, Ospedale Maggiore Policlinico Foundation Milano, Italy; 6 Department of Informatics, Systems and Communication, University of Milano-Bicocca, Milano, Italy; 7 Mosaiques Diagnostics GmbH, Hannover, Germany; 8 Center for Proteomics and Metabolomics, Leiden University Medical Center, Leiden, The Netherlands; Università degli Studi di Milano, Italy

## Abstract

Renal Cell Carcinoma (RCC) is typically asymptomatic and surgery usually increases patient's lifespan only for early stage tumours. Moreover, solid renal masses cannot be confidently differentiated from RCC. Therefore, markers to distinguish malignant kidney tumours and for their detection are needed. Two different peptide signatures were obtained by a MALDI-TOF profiling approach based on urine pre-purification by C8 magnetic beads. One cluster of 12 signals could differentiate malignant tumours (n = 137) from benign renal masses and controls (n = 153) with sensitivity of 76% and specificity of 87% in the validation set. A second cluster of 12 signals distinguished clear cell RCC (n = 118) from controls (n = 137) with sensitivity and specificity values of 84% and 91%, respectively. Most of the peptide signals used in the two models were observed at higher abundance in patient urines and could be identified as fragments of proteins involved in tumour pathogenesis and progression. Among them: the Meprin 1α with a pro-angiogenic activity, the Probable G-protein coupled receptor 162, belonging to the GPCRs family and known to be associated with several key functions in cancer, the Osteopontin that strongly correlates to tumour stages and invasiveness, the Phosphorylase b kinase regulatory subunit alpha and the SeCreted and TransMembrane protein 1.

## Introduction

Biomarkers able to characterize and predict multifactorial diseases, such as cancer, are still one of the most important targets for all the “omics” investigations.

These clinically oriented studies have also been successfully performed in the peripheral fluids, taking advantage of non- or very low-invasive collection methods. In particular, the urinary low-molecular-weight proteome, also termed urinary peptidome [1], [2], represents an important source of information for biomarker discovery.

The analysis of the urinary peptidome should be most applicable to renal diseases, given that urine should contain a higher amount of molecules, including these naturally occurring polypeptides, with an altered concentration deriving directly from kidney. In particular, Renal cell carcinoma (RCC) needs markers for detection, prognosis and therapeutic targeting [3]. Whereas RCC includes an heterogeneous group of tumours with variable clinical outcomes, that range from indolent to explicitly malignant [4], the most common histological type is represented by clear cell RCC (ccRCC), and comprises approximately 60% of all renal tumours [5].

RCC is the third most frequent malignancy of the genitourinary tract accounting for about 90% of all renal malignancies and the most fatal urological cancer, causing approximately 2% of all cancer deaths [6]. It is noteworthy that this carcinoma is one of the human cancers with an increasing incidence. Currently, as RCC is typically asymptomatic, most cases are frequently detected as an incidental renal mass, imaging the abdomen for other reasons such as during the work-up of acute renal failure [5]. About 30% of RCC patients will present metastases at the time of the diagnosis, many others will develop metastasis after surgical resection and for these patients the prognosis is dismal. Indeed, treatment of metastatic RCC remains highly challenging since its progression-free survival is very poor among these patients [3]. Traditionally RCC is known to be refractory to chemotherapy and to radiotherapy. Surgical removal of the tumour is considered the only effective treatment and, where feasible, may result in remission in up to 40–60% of cases [7].

Management of RCC is benefiting from the increasing role of small tumour masses detection, greater understanding of the metabolic pathways involved, new targeted medical treatments for metastatic RCC, and evolving surgical and minimally invasive image-guided treatment techniques [8]. Although in the absence of biomarkers, renal imaging is most often recommended by advocates of screening, a confident histological classification and diagnosis with this technique is not always feasible, especially for some ambiguous cystic and solid renal lesions [9]. Therefore, early diagnosis could highly improve survival rates for patients with renal cancer and also for those with localized tumours. Moreover, welfare will benefit from a test able to distinguish small kidney malignant masses from benign lesions driving the patient to low or high intense follow-up.

The present work is focused on the application of a single-step purification using C8 functionalized magnetic beads (C8-MB) followed by MALDI-TOF analysis and nLC-ESI-MS/MS to explore possible urinary peptide signatures of patients affected by ccRCC, by other kidney tumours and control subjects.

## Materials and Methods

### Chemicals and standards

Profiling Kit 1000 C8-MB, α-cyano-4-hydroxycinnamic acid (CHCA), Protein Calibration Standard I (ProtMix I) and Peptide Calibration Standard II (PepMix II) were supplied by Bruker Daltonics GmbH (Bremen, Germany).

### Urine collection and handling procedure

Urine samples were collected from patients the day before surgery and from healthy volunteers at “Ospedale Maggiore Policlinico” Foundation (Milan, Italy), San Gerardo Hospital (Monza, Italy) and Desio Hospital (Desio, Italy). All subjects had signed an informed consent prior to sample donation. Study protocols and procedures were approved by the local ethic committee (U.O. Comitato di Etica e Sperimentazione Farmaci Direzione Scientifica Fondazione IRCCS Ca'Granda Ospedale Maggiore Policlinico, Milano and Comitato Etico Azienda Ospedaliera San Gerardo, Monza) and analysis were carried out in agreement with the Declaration of Helsinki. Second morning midstream urine was collected in sterile urine tubes (Anicrin s.r.l., Italy) [10].

As concerning the MB protocol, the binding, washing and desorption steps of the beads were based on the manufacturer instructions and slightly modified, as previously reported (see supplemental material S1) [11]. In particular, 40 µL of urine of each subject was used both for MALDI-TOF profiling and the preparation of two urine pools (n = 80 each) used for peptide identification. The two pools from ccRCC patients or control subjects urine were manually purified with magnetic beads.

### MALDI-TOF peptide profiling

Fractionated samples were analysed in linear mode (MALDI-LM) and reflector mode (MALDI-RM) using an UltrafleXtreme™ MALDI-TOF/TOF instrument (Bruker Daltonics, Germany) as previously described and reported in supplemental materials S1 [11]. Spectra processing was based on baseline subtraction and realignment using a subset of seven frequent common peaks (at m/z-values 1162, 1511, 1681, 1895, 1912, 2236, and 3373) for liner mode with a peak tolerance of 1000 ppm. An internal calibration was also performed on reflector data using four peaks (at m/z-values 1680.93, 1912.06, 2040.17 and 2659.32) with a tolerance in peak assignment of 100 ppm except for the last one that was 200 ppm.

ClinProTools™ software v. 2.2 (Bruker Daltonics, Germany) was used for multiple spectra comparison after their normalization. Data selection was performed with: resolution value of 800, “Convex Hull” baseline correction with a baseline flatness of 0.80, null or not recalibratable spectra exclusion. The mean spectrum obtained from each subject data set was used for the statistical elaboration.

List of peaks (m/z) with their area (as a measure of compound abundance) was obtained with a S/N threshold of 3 and peak areas were calculated using zero level integration type on the total average spectrum for the statistical analysis.

Mean area values, before and after spectra processing, of all clusters signals for each sample groups are reported in **Table S1**.

### Peptide identification by MALDI-TOF/TOF

For peptide identification, LIFT-TOF/TOF spectra were acquired using the UltrafleXtreme™ MALDI-TOF/TOF mass spectrometer without additional collision gas. Analyses were performed using appropriate acquisition settings as previously reported [11]. MS/MS data were processed using FlexAnalysis™ software v. 3.3 (Bruker Daltonics, Germany). Database searching was performed by an in-house Mascot search engine (Version: 2.4.1) with the same parameters already described [11].

### Expression profile analysis and statistical analysis

Statistical analysis was conducted following the sequence of processes as reported in **Figure S1** and specified in its caption. Briefly, to apply the correct statistical procedure we first evaluated the assumption of normality and the homogeneity of variance, then appropriate parametric or non-parametric tests were used for case/control comparisons and correlations. Discriminant models were built using Rapid Miner (RaM) [12]–[14] workflow based on SVM algorithm (**Figure S2** and its caption). The performances of our inference process *a*re given through indices which are broadly applied to measure the *classification performance* of an inference system; *i.e.*, sensitivity, specificity, positive (PPV) and negative predictive values (NPV) [15]. The receiver operating characteristic curve analysis (ROC) and area under the curve (AUC) were also evaluated as previously described [16].

### Peptide identification by nLC-ESI-MS/MS

Endogenous peptides in the enriched fractions obtained by MB purification of urine pools from controls (n = 80) and ccRCC patients (n = 80) were identified by nLC-ESI-MS/MS. Briefly, purified samples were desalted using Ziptip™ µ-C18 Pipette Tips (Millipore Corp, Bedford, MA) as already reported [11]. Desalted fractions were injected into Dionex UltiMate 3000 rapid separation (RS) LC nano system (Thermo Scientific, Germany) coupled online with an Impact HD™ mass spectrometer (Bruker Daltonics, Germany).

Peptides were first loaded onto a µ-precolumn (Dionex, Acclaim PepMap 100 C18, cartridge, 300 µm i.d.×5 mm, 5 µm), followed by separation on the analytical 50cm nano column (Dionex, 0.075 mm ID, Acclaim PepMap100, C18, 2 µm). Multistep 360 min gradients with a ramp from 4 to 35% in 245 min of mobile phase B (0.1% FA/80% CHCN) were used. The mass spectrometer was operated in the data-dependent-acquisition mode.

Raw MS/MS data were lock-mass corrected, deconvoluted and converted to XML peak lists and processed using an in-house Mascot search engine (v2.4.1). Peptide identification details are provided in Supplemental Materials S1. Briefly, database searching was restricted to human Swiss-Prot (accessed Apr 2014, 544,996 sequences; 193,815,432 residues). No enzyme and any fixed modification was set in search parameters. Mass tolerances for all identifications were generally fixed at 20–5 ppm MS and 0.5–0.05 Da MS/MS. Acetyl (N-term) was set as variable modification in Mascot search parameters. Mascot thresholds score for homology and identity and decoy database were used as peptide level filters of peptide significance (False Discovery Rate <1%).

## Results

### Clinical data and study design

Urine collected from 137 healthy subjects (Ctrls) (81 men, 56 women), 118 clear cell RCC (ccRCC) (73 men, 45 women), 35 other different histological subtypes (non-ccRCC), (22 men, 13 women) (16 benign renal masses and 19 malignant non-ccRCC) patients were used in the present study. Fisher test did not reveal any gender dependence in the studied cohort. Mean age for controls was 48.7 with a range of 24–79 years, while 64.53 for patients with a range of 33–88 years. Patients were classified according to the 2009 TNM (tumour-node-metastasis) system classification and their clinical characteristics are described in **Table 1** [17]. Histological analysis was performed on patients based upon the Fuhrman grading system, sarcomatoid and cystic differentiation, tumour necrosis, microvascularity and urinary infiltration. Tumour patients underwent surgical excision of the renal lesion.

**Table 1:** Patients clinical characteristics according to the 2009 TNM (tumour-node metastasis) system classification.

	N° of PATIENTS
**ALL**	153
Mean ± SD age at diagnosis	64.53±10.97
Median age at diagnosis (range)	33–88
**GENDER**	
Males	95
Females	58
**STAGING**	
Primary Tumor (T)	
* pT1*	91
* pT2*	26
* pT3*	12
* pT4*	0
* unknown*	8
Regional lymph nodes (N)	
* NX*	77
* N0*	40
* N1*	1
* unknown*	19
**GRADE**	
G1	7
G2	86
G3	24
G4	3
* unknown*	17
**HISTOLOGY**	
Clear cell RCC	118
Papillary RCC	9
Chromophobe	5
Oncocytoma*	8
Angiomyolipoma*	5
Other subtypes**	8
**TUMOR TYPE**	
Malignant	137
Benign	16

*  =  benign renal masses

** = 5 malignant subtypes and 3 benign renal masses

### Protein profiles and cluster analysis

Aiming to perform a profiling analysis of the urinary peptidome and build statistical patterns of potential discriminant biomarkers, group comparisons were evaluated.

About 202 peaks common to the three groups have been detected in MALDI-TOF averaged peptide profiles of controls, ccRCC and non-ccRCC patients after C8-MB sample pre-purification. Ion signals correlating with the age (Spearman test) were not considered in the subsequent elaborations [18].

Discriminant pattern recognition was performed using SVM algorithm supplied by RaM software. Initially, the entire cohort of patients was divided into malignant tumours (n = 137) and benign renal masses plus healthy subjects (n = 153) and a discriminant cluster of twelve peptides (linear mode *m/z*-values 1116, 1670, 2216, 2528, 2661, 3162, 3443, 5032, 5532, 6130, 6786 and 10654) was built with 78% and 88% of specificity and sensitivity, respectively, (**Table 2A**).

**Table 2:** Performances of the cluster of twelve signals to discriminate malignant tumours from benign renal masses or controls (False  =  Benign or controls; True  =  Malignant) with *k-fold*  = 10 cross-validation (A) and of the model, originated in the training phase using about 60% of the data, in validation test using the other about 40% of the studied subjects (B).

**A**	true False	true True	class precision
pred. False	120	17	87.6%^a^
pred. True	33	120	78.4%^b^
Spec., Sens.	78.40%	87.60%	
**B**	true False	true True	class precision
pred. False	53	13	80.3%^a^
pred. True	8	42	84.0%^b^
Spec., Sens.	86.90%	76.40%	

Spec. =  Specificity; Sens. =  Sensitivity; pred.  =  Prediction; Class precision: a =  Negative Predictive Value and b = Positive Predictive Value; Precision  =  Relative number of correctly classified examples among all examples classified as positive i.e. precision = (Positives Correctly Classified)/(Total Predicted Positives). Note that the Total Predicted Positives is the sum of True Positives and False Positives. This is the same for the Negative Predictive Value. True True  =  True positive; true False  =  True negative

Based on the recommendations for biomarker discovery and qualification in clinical proteomics [19], the model was subsequently evaluated in an independent set of 61 (benign and controls) and 55 (malignancies) subjects (**Table 2B**). The cluster of 12 signals allowed us to discriminate the benign/controls from malignant tumours with 87% and 76% of specificity and sensitivity respectively, and an AUC of 0.89 (**Figure S3**), confirming its high diagnostic accuracy according to the criteria suggested by Swets [20]. Peaks selected by SVM to build the discriminant clusters have not necessarily to be statistically different in the group comparisons. They are selected through a forward selection scheme with SVM as inference procedures which, in turn, follows a different way to select discriminative features. Statistical analysis showed that eight of these peaks (at *m/z*-values 1116, 1670, 2216, 2528, 2661, 3162, 3443 and 5532) had a higher urinary concentration (p<0.05) while three (at *m/z*-values 5032, 6130 and 6786) had a lower urinary abundance (p<0.05) in malignant kidney tumour patients compared to controls and subjects with benign renal masses (**Table 3** **and Figure S4**).

**Table 3:** Urinary relative concentration (malignant/benign+ctrl) and p-value of the twelve ions included in the model able to distinguish benign renal masses or controls from malignant kidney tumours.

Ions (m/z)	p-value	Urinary concentration
1116	<0.001	Up
1670	<0.001	Up
2216	<0.05	Up
2528	<0.001	Up
2661	<0.001	Up
3162	<0.001	Up
3443	<0.001	Up
5032	<0.001	Down
5532	<0.05	Up
6130	<0.001	Down
6786	<0.001	Down
10654	> 0.05	n.s.

n.s.  =  not statistically different.

In addition, using an interactive analysis, a different case/control classification task was also performed for Ctrls versus ccRCC. Classification performances in training and validation test are reported in **Table 4**. All controls and ccRCC patients were used for feature selection and cross-validation procedures and a cluster of twelve peptides (linear mode *m/z*-values 1670, 1727, 2192, 3005, 3252, 3636, 4623, 5432, 5532, 5964, 6062 and 6175) was selected by a statistical analysis of MALDI-TOF spectra. Performance of the model resulted in a specificity of 90% and in a sensitivity of 82% (**Table 4A**). This good diagnostic capability was proved by a validation on the second independent set of data (53 Ctrls and 49 ccRCC patients), confirming the specificity of 91% and a sensitivity of 84% (**Table 4B**). Four ions (at *m/z*-values 4623, 5432, 6062 and 6175) were observed to be in lower concentration (p<0.05) in ccRCC patients, and other four (at *m/z*-values 1670, 1727, 3636 and 5532) showed an increased abundance (p<0.05) (**Table 5** **and Figure S5**). The AUC of the model was 0.96 (**Figure S6**), pointing towards an accurate test [20].

**Table 4:** Performances of the cluster of twelve signals to discriminate ccRCC patients from controls (False  =  Controls; True  =  ccRCC) with *k-fold*  =  10 cross-validation (A) and of the model, originated in the training phase using about 60% of the data, in validation test using the other about 40% of the studied subjects (B).

**A**	true False	true True	class precision
pred. False	123	21	85.42%^a^
pred. True	14	97	87.39%^b^
Spec., Sens.	89.78%	82.20%	
**B**	true false	true true	class precision
pred. false	48	8	85.71%^a^
pred. true	5	41	89.13%^b^
Spec., Sens.	90.57%	83.67%	

Spec.  =  Specificity; Sens.  =  Sensitivity; pred.  =  Prediction; Class precision: a  =  Negative Predictive Value and b  =  Positive Predictive Value. Precision  =  Relative number of correctly classified examples among all examples classified as positive i.e. precision  =  (Positives Correctly Classified)/(Total Predicted Positives). Note that the Total Predicted Positives is the sum of True Positives and False Positives. This is the same as the Negative Predictive Value.

True True  =  True positive; true False  =  True negative.

**Table 5:** Urinary relative concentration (ccRCC/ctrl) and p-value of the twelve ions included in the model able to distinguish controls from ccRCC.

Ions (m/z)	p-value	Urinary concentration
1670	<0.001	Up
1727	<0.001	Up
2192	> 0.05	n.s.
3005	> 0.05	n.s.
3252	> 0.05	n.s.
3636	<0.001	Up
4623	<0.001	Down
5432	<0.001	Down
5532	<0.05	Up
5964	> 0.05	n.s.
6062	<0.001	Down
6175	<0.001	Down

n.s.  =  not statistically different.

### Endogenous peptide identification

The identification of urinary endogenous peptides corresponding to signals included in the two discriminative models was obtained by nLC-ESI-MS/MS analysis of C8-MB enriched fractions (two pools of 80 controls and 80 patients). Alignment of the *m/z*-values determined by MALDI-LM with those from LC-ESI was based on the mass spectra acquired by MALDI-RM of the C8-MB fractions. The mass measurement errors (MMEs) of assigned peptides measured by MALDI-RM and by MALDI-LM varied from −53 to 72 ppm and −297 to 77 ppm, respectively. The differences between mass measurements in ESI and MALDI-RM mode varied from −79 to 50 ppm. Seven MALDI-LM signals were assigned to eight different protein fragments (**Table 6**).

**Table 6:** Identification of seven MALDI signals included in the discriminant clusters.

MALDI			nLC-ESI MS/MS										
Relative Abundances	LM *m/z*	RM *m/z*	Theoretical Monoisotopic Mr + [H+]	Theoretical Average Mr + [H+]	Experimental Mr + [H+]	Charge	Uniprot ID	MW (Da)	Start	Sequence	Stop	PTM	Pep_score
↑ in malignant	1116	1115.60	1115.559	1116.209	1115.551	2	SCTM1_HUMAN	27039	69	RAHGQESAIF	78		58
↑ in ccRCC ↑ in malignant	1670	1668.84	1668.928	1669.899	1668.923	2	UROM_HUMAN	69761	590	GSVIDQSRVLNLGPIT	605		32
↑ in ccRCC	1727	1725.92	1725.877	1726.865	1725.872	2	MEP1A_HUMAN	84419	636	EEALPVSLSQGQPSRQ	651		88
n.s.	2192	2190.89	2190.771	2192.179	2190.765	4	GP162_HUMAN	63930 (Isoform 1)	344	IMSEEDGDDDGGCDDYAEGR	363	N-acetylation	26
								33062 (Isoform 2)					
			2190.913	2192.253	2190.778	5	KPB1_HUMAN	137312	1021	AESQSPGTSMTPSSGSFPSAYD	1042		22
↑ in malignant	2216	2215.39	2215.231	2216.523	2215.214	4	UROM_HUMAN	69761	587	FRSGSVIDQSRVLNLGPITR	606		54
↑ in malignant	2528	2527.26	2527.231	2528.706	2527.217	4	OSTP_HUMAN	33017–35423	19	VKQADSGSSEEKQLYNKYPDAVA	41		55
↑ in malignant	2661	2659.44	2659.257	2660.751	2659.252	5	FIBA_HUMAN	94973 (Isoform 1)	605	DEAGSEADHEGTHSTKRGHAKSRPV	629		39
								69757 (Isoform 2)					

The assignment was performed through an accurate mass alignment with peptides identified by nLC-ESI MS/MS from C8-MB enriched urine samples. Mascot peptide score (pep_score), molecular weight of the related intact protein or protein isoform (MW), possible hypothetical modification predicted by Mascot (PTM) and the relative abundances of each peptide (p < 0.05) in ccRCC (n = 118) *vs* controls (n = 137), and/or in malignant tumours (n = 137) and benign renal masses plus healthy subjects (n = 153) have been reported.

↑  =  over-represented; ↓  =  under-represented; n.s.  =  not statistically different; LM  =  linear mode; RM  =  reflector mode.

The identity of the peptide giving rise to the MALDI-LM signal at m/z 2192, included in the model, could not be unambiguously obtained (**Table 6**). The specific contributes to the MALDI-LM peak of the two peptides at m/z 2190.765 (GP162) and at m/z 2190.778 (KPB1), identified by LC-ESI, were prevented from being distinguished due to the low molecular mass differences between the two amino acid sequences coupled with the resolution power of MALDI-RM (**Table 6**).

The identity of one signal present in MALDI-LM spectra corresponding to *m/z*-value 2661, assigned to a fragment of FIBA, was further confirmed by MALDI-TOF/TOF (Mascot score exceeding the significant threshold of identity, score = 166, p<0.05).

## Discussion

To date, thousands of different proteins/peptides have been sequenced in human urine, providing a greater insight into the urinary content and suggesting more exhaustive disease-specific researches for their potential use in clinical practice. Biomarker discovery studies are widely performed on urine samples, with the aim of developing a non-invasive diagnostic tool for prostate cancer [21], diabetic nephropathy [22], chronic kidney disease [23] as well as for RCC [3], [10], [24].

Thus far, many studies performed using Western blot analysis have reported several proteins with an altered urinary concentration in RCC patients, relative to control subjects, with potential diagnostic/prognostic capabilities. An over-concentration of the urinary nuclear matrix protein 22 was found in 23 of 35 RCC patients compared to 30 patients with kidney stone and renal cystis used as controls [25]. The urinary 14-3-3 protein alpha/beta has also been shown to be in a higher concentration in RCC patient urine compared to that from healthy volunteers samples [26]. The diagnostic capability of this protein resulted in an AUC of 0.88. Two other proteins, Aquaporin-1 and Peirilin-2, were observed to be at higher levels in the urine of 63 RCC patients versus 43 healthy subjects [27]. The sensitivity and specificity values were in the range of 90–100% for both these proteins. The urinary concentration of these proteins returned to levels similar to those found in controls 2–4 weeks after surgery. KIM-1, a biomarker for the detection of proximal tubules epithelial cells after ischemic or toxic injury in humans, has also been shown to be increased in the urine of RCC patients [28]. However, none of these results have been validated in an independent cohort of patients.

Highly sensitive profiling studies require a combination of MS and separation technologies based on different types of chromatography. Recently, Frantzi *et al.* have described a model of 86 signals detected in the urine of healthy subjects and RCC patients by CE-MS with a sensitivity and specificity of 80% and 87%, respectively [29]. They could identify 40 of these markers and most of them were fragments of collagen, fibrinogen and Na/k-transporting ATPase. The authors suggest that these markers are the consequence of different proteases specific to RCC, of changes in proteolytic activity in the microenvironment of the kidney tumours, and of kidney damage.

Successful discoveries of peptide signatures through MALDI MS profiling have been reported for various human diseases comprising of kidney pathologies [10]. In these studies urine samples were pre-fractionated before MS-analysis through different approaches [30] or beads [31]–[34].

Aiming to detect clusters of ions with diagnostic capability in RCC, urinary protein profiles were investigated by several groups using SELDI technology. Rogers *et al.* were able to build a neural-network model with a sensitivity and a specificity of 98.3–100%. These performances declined to 41–77% when tested in an independent set of samples [30]. Later, Wu *et al.* also built a cluster with four differentially represented peptides showing a sensitivity and specificity of 80%–89.6% in the training phase, that decreased to 67.8–81.5% in the validation phase [35]. However, none of these groups were able to provide the identity of their markers. Recently Alves *et al.* [36] could cluster RCC patients and controls using a SELDI-TOF approach. They were also able to provide the identity of several protein signals by SDS-PAGE followed by LC-ESI but no information about the diagnostic capability of the outcomes was given. We have previously reported in a pilot study the possibility to discriminate kidney tumours (34 clear cell RCC, 4 papillary RCC and 1 mixed RCC+papillary) from controls based on urinary signature [10]. However, due to the low and not homogenous groups of patients and controls, results have to be confirmed on a wider and more appropriate dataset. Moreover, the possibility to differentiate malignant from benign renal masses has not be considered.

Therefore, in this study we have investigated the urinary low molecular weight proteome in a larger cohort of healthy volunteers (n = 137), ccRCC (n = 118), and of 35 non-ccRCC patients using a pre-purification procedure based on C8 functionalized magnetic beads in combination with MALDI-TOF analysis. About sixty percent of the enrolled patients and controls were used in the training phase of the SVM and the remaining subjects for the independent test evaluation of the models. Patterns of urinary peptide markers able to distinguish RCC from controls and to significantly differentiate kidney cancer from benign lesions were searched.

In biomarker discovery and classification field, the size of the model is a crucial aspect [37]. The model should be built avoiding over-fitting but preserving generalization, in terms of capability to correctly classify new subjects. In this study we limited the clusters to no more than 20 features [37].

Initially we focused on the possibility to distinguish benign renal masses or healthy subjects from malignant tumours and a classifier with twelve urinary peptides with an AUC of 0.89 was generated. Then, we afforded the possibility to discriminate ccRCC patients from healthy subjects and a classifier with twelve peptides was selected with good discriminating capability that were confirmed in an independent cohort of subjects with an AUC of 0.96. Identity of seven of the ions included in the clusters were obtained by MALDI-TOF/TOF and by nLC-ESI-MS/MS analysis. Most of them were different from those identified by CE-MS and used in the model by Frantzi *et al.* [29] and, interestingly, most of them were correlated to the presence of a tumour mass. This is not surprising since the data on urinary peptidome delivered from different pre-fractionation of sample and from a different chromatographic separation provide complementary information [38].

Hereby we describe two patterns of twelve urinary peptides with a high discrimination power obtained by an SVM-based statistical approach. Seven of these signals were most likely identified.

In particular, two ions at *m/z* 1670 and 2216 observed in MALDI-LM spectra were identified as fragments of the human glycoprotein uromodulin (UMOD/THP) and they were present in higher concentration in patients affected by both ccRCC and other malignant kidney tumours. The urinary excretion of UMOD has been studied in various physio-pathological states, but its precise biological role is still undefined. Clinical relevance of this protein has been described in several pathologies and THP mutations have been associated with chronic kidney disease, altered glomerular filtration rate and decreased urinary excretion [39]. Furthermore, decreased UMOD expression has been observed in end-stage renal disease, in kidney neoplasms [40], [41] and in cysts from autosomal dominant polycystic kidney disease [42]. Moreover it was also reported with a lower abundance in other pathologies like renal calculi disease [43], IgA nephropathy [44] or diabetic nephropathy [45]. The relative concentration of two urinary UMOD fragments, at *m/z* 1912 and 1824, included in a discriminant model able to distinguish RCC patients from controls in our previous pilot study [10], was confirmed by our findings (data not shown). In a peptidome profiling study on urine samples from healthy subjects exposed to high altitude hypoxia another UMOD peptide, Val_592_IDQSRVLNLGPITArg_606_, a few amino acids shorter than fragments identified in this study, was also detected as altered in urinary levels [34].

The ion at *m/z* 2659 was identified as a fragment of fibrinogen alpha chain (FIBA) and was found highly represented in the urine of patients with cancer compared to healthy subjects. This peptide was also identified by Siwy J *et al.* [46] but not included in their discriminative model for RCC [29].

However, particular significance could be ascribed to those proteins, showing a strong correlation with tumour development and progression. A fragment of MEP1A (*m/z* 1727), a zinc-dependent metalloproteinases abundantly expressed in the apical membranes of renal proximal tubules was observed as over represented in ccRCC urine [47], [48]. It has been recently reported that MEP1A enzyme exhibits a broad expression pattern, implicating functions in angiogenesis, cancer, inflammation and fibrosis [49]–[52]. Interestingly, a relevant pro-angiogenic activity has been described for this meprin [53], [54] with a molecular mechanism based on proteolytic activation of pro-angiogenic growth factors, such as VEGF-A [55]–[57]. Moreover, meprin α is reported to be expressed in several different tumours, as in breast and colorectal carcinomas [58], [59] and probably associated to the transition to malignant stages of colorectal carcinoma [54]. However, its onco-expression is likely to be specific between different cancers, *e.g.* with quite low levels in ovarian cancer compared with gastrointestinal carcinomas [60]. Finally, there is data indicating that meprins are involved in a complex with hypoxia-inducible factor-1a (HIF-1a) proposing a possible participation of these proteins in oxygen sensing mechanisms and in the response of the kidney proximal tubule cells to hypoxia process [61].

Two different amino acid sequences have been recognized to give rise to the ccRCC discriminant signal observed at m/z 2192 in MALDI-LM spectra: a fragment of Probable G-protein coupled receptor 162 (GP162) and a sequence derived from Phosphorylase b kinase regulatory subunit alpha, skeletal muscle isoform (KPB1). The first protein (GP162), is an orphan receptor assigned to G protein coupled receptors (GPCRs) family involved in signal transmission [62]. GPCRs are associated with several functions largely correlated to cancer such as cell proliferation, angiogenesis, tumour progression and development [63]. Many GPCRs are over-expressed in various cancer types and they are constitutively active in malignant cells causing an aberrant response to various signals [63]. The protein Phosphorylase b kinase regulatory subunit α is a key regulatory enzyme of glycogen metabolism [64]. Glycogen can be broken down rapidly when glucose is needed, and Phosphorylase b kinase switches on another enzyme called glycogen phosphorylase b by converting it into the more active form, glycogen phosphorylase a. Alteration of KPB1 seems to be associated with muscle phosphorylase b kinase (PHK) deficiency, a rare disorder caused by mutations in the gene coding for this protein [65]. To our knowledge, whereas this protein certainly plays an important role in providing energy for cells, there is no evidence in literature that may explain a possible association with cancer.

A fragment of OSTP (*m/z* 2528) was found in higher concentration in urine of malignant tumour patients. Several studies have shown its abundance both in tumour and tumour microenvironment cells [66]. In particular, a significant cytoplasmic staining has been reported for a variety of cancer tissues, including renal carcinomas, while a low staining has been shown for breast and skin cancers [67]. It was proposed a correlation between tissue and plasma/serum levels of Osteopontin and prognosis in a huge number of cancers. In particular, Coppola *et al*. have suggested a possible role of Osteopontin in tumour progression as they found a high expression of OSTP in 72% of tissue samples of RCC patients analysed by immunohistochemistry and these results correlated with tumour stage [67]. In addition, Ramankulov *et al.* analysed plasma of RCC patients at different stages and metastatic grades by an enzyme immunometric assay and they described high levels of this protein in plasma of patients with RCC with regional lymph nodes and its expression reached the highest values in patients with distant metastases [68]. Ye *et al.* analysed urine samples of postmenopausal women with ovarian cancer and benign conditions and from nonsurgical controls by SELDI-TOF MS and two-dimensional gel electrophoresis and they identified some fragments of Osteopontin strongly correlated to tumour stages and invasiveness, suggesting their use as urine biomarkers, in particular for patient prognosis and tumour metastatic power [69]. Furthermore, the high expression of Osteopontin and CD44 in tissue samples of ccRCC seems to correlate with poor prognosis [70].

A fragment of the extracellular domain of SeCreted and TransMembrane protein 1 (SCTM1) (*m/z* 1116) was at a higher intensity in the urine of patients affected by kidney malignant tumours. The SECTM1 protein level was observed to be increased in many tumours, including breast cancer, leukemia cell lines and melanoma [71]. Kuk *et al.* have included SCTM1 among a panel of 52 possible candidate biomarkers for ovarian cancer [72].

In conclusion, the discriminant models described in this study might be useful in distinguishing renal masses which can't be confidently defined using radiology alone and, as it stands, still need a confirmatory biopsy. In compromised patients, kidney biopsies can be technically challenging and hazardous. These patients would benefit from our findings, avoiding this invasive procedure in order to attain a correct diagnosis. Moreover, the urinary proteomic signals typical of kidney cancer could also be extremely useful for the evaluation of the vitality of cancer cells, during and after target therapies, to estimate the response to the treatment. Urine markers could also help to detect initial relapses in partially resected kidneys that are often very hard to diagnose as current imaging techniques are unable to easily distinguish between a surgical modification of the parenchyma, due to a inhomogeneous scar, or an initial disease recurrence. Furthermore, urinary markers could easily assess the residual vitality of the tumour after minimally invasive techniques, such as ablation with percutaneous radio-frequency, cryotherapy, microwave and high-intensity focused US (HIFU), which are suggested as treatments in selected patients with a genetic predisposition to multiple tumours, with a solitary kidney, with bilateral tumours or elderly patients. Since further research is needed in order to determine the oncological success rate of these procedures and due to the efforts related to the follow-up, urinary markers may be used to survey these patients.

## Supporting Information

Figure S1 **Scheme of the statistical analysis using R and RapidMiner.** Differentially represented signals are detected (block 5) according to the standard assumptions for parametric models (block 3). RapidMiner (RaM) was applied (block 6) for forecasting a suitable predictive cluster of signals. The conceptual sequence of operational steps applied in RaM is given in Figure S2.
(TIF)

Figure S2 **RapidMiner workflow.** A: Data is retrieved by the “Input” operator and the feature selection is performed (“Forward Selection operator”). B: Feature selection encapsulates a cross validation process (“Cross Validation operator”) to select the most performing set of features. C: Cross Validation operator encapsulated a *k-fold* cross validation process. First a classifier is built describing a predetermined set of data classes. Then, the model (a trained SVM) is used for testing new classification examples. The first inner operator (“SVM”) realizes the first step (Training). The second inner operator (“Apply Model”) realizes the second step. Finally, the predictive accuracy of the classifier is estimated by the “Performance” operator (Testing). Blocks 4 and 5 in panel A are given to provide ROC curve analysis (e.g. see Figure S3 for malignant *vs* benign plus controls and Figure S6 for controls *vs* ccRCC).
(TIF)

Figure S3 **ROC curve analysis of the model discrimination performance when applied to an independent cohort of subjects (malignant** ***vs*** **benign/ctrl).** ROC curves are calculated by first ordering the classified examples by confidence. Afterwards all the examples are taken into account with decreasing confidence to plot the false positive rate on the x-axis and the true positive rate on the y-axis. The threshold (blue line) refers to the confidence value of the prediction, i.e. if the confidence of the example to be positive is greater than the threshold, the example will be classified as positive, if the confidence is below the threshold, it will be classified as negative.
(TIF)

Figure S4 **Box-plot of the eleven ions included in the model able to distinguish benign or controls from malignant tumours and statistically different (p<0.05) in the two groups (see** **Table 3** **). Y-axis refers to arbitrary intensity.**
(TIF)

Figure S5 **Box-plot of the eight ions included in the model able to distinguish controls from ccRCC and statistically different (p<0.05) in the two groups (see** **Table 5** **). Y-axis refers to arbitrary intensity.**
(TIF)

Figure S6 **ROC curve analysis of the model discrimination performance when applied to an independent cohort of subjects (controls** ***vs*** **ccRCC).** The threshold (blue line) refers to the confidence value of the prediction, i.e. if the confidence of the example to be positive is greater than the threshold, the example will be classified as positive, if the confidence is below the threshold, it will be classified as negative.
(TIF)

Table S1 **Mean area values of the signals included in the two clusters discriminating malignant tumours from benign renal masses plus controls (A) and ccRCC from controls (B) calculated from raw data before and after spectra elaboration.**
(DOCX)

Materials S1 **Details of the experimental protocols.**
(DOCX)
