# Supplementary figures and images for: Urinary Signatures of Renal Cell Carcinoma Investigated by Peptidomic Approaches
Source: PLoS One. 2014 Sep 9;9(9):e106684. doi: 10.1371/journal.pone.0106684 (PMC4159280; doi:10.1371/journal.pone.0106684)

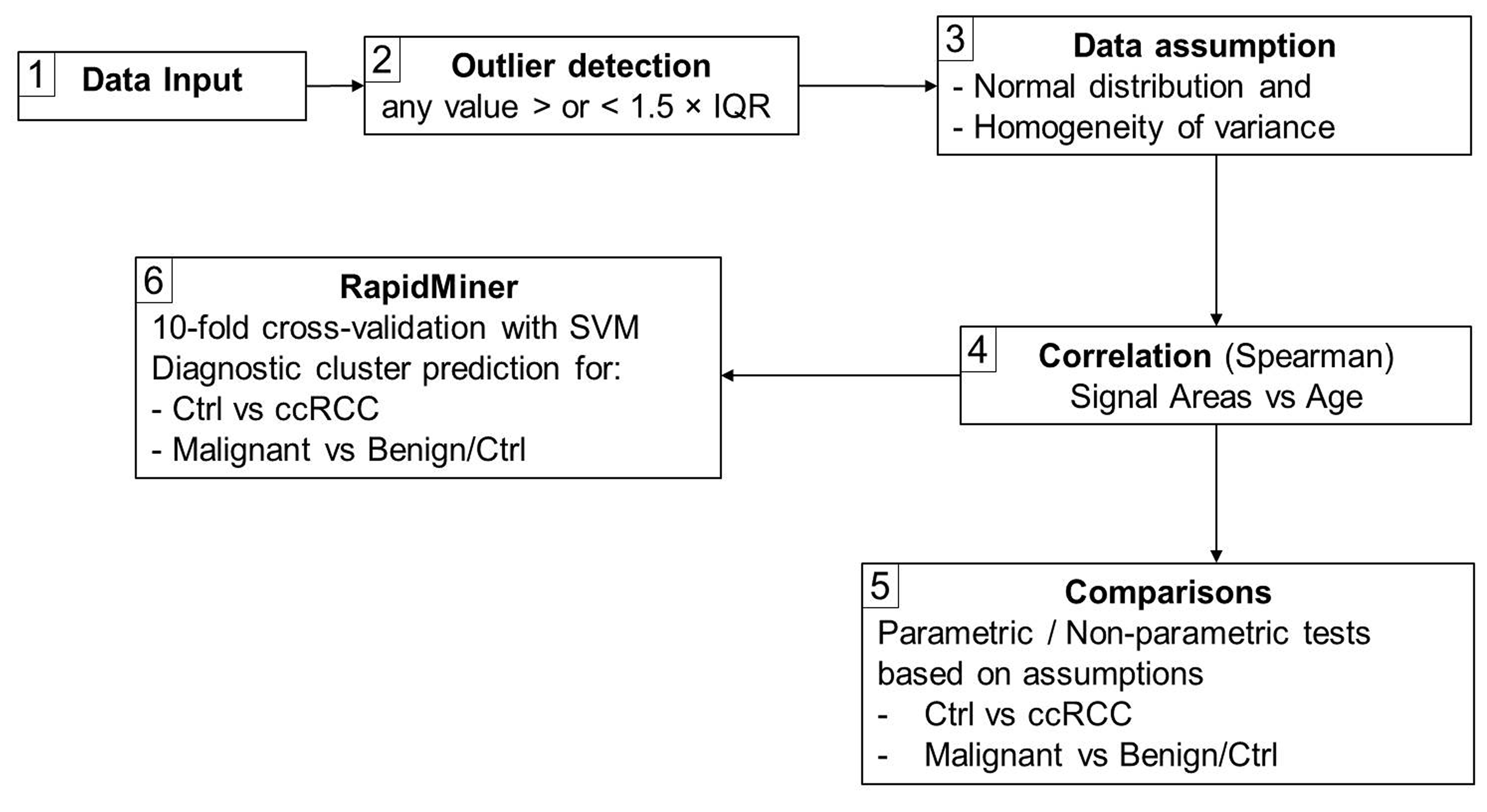

Supplement: Figure S1 — Scheme of the statistical analysis using R and RapidMiner. Differentially represented signals are detected (block 5) according to the standard assumptions for parametric models (block 3). RapidMiner (RaM) was applied (block 6) for forecasting a suitable predictive cluster of signals. The conceptual sequence of operational steps applied in RaM is given in Figure S2. (TIF) [file pone.0106684.s001.tif]

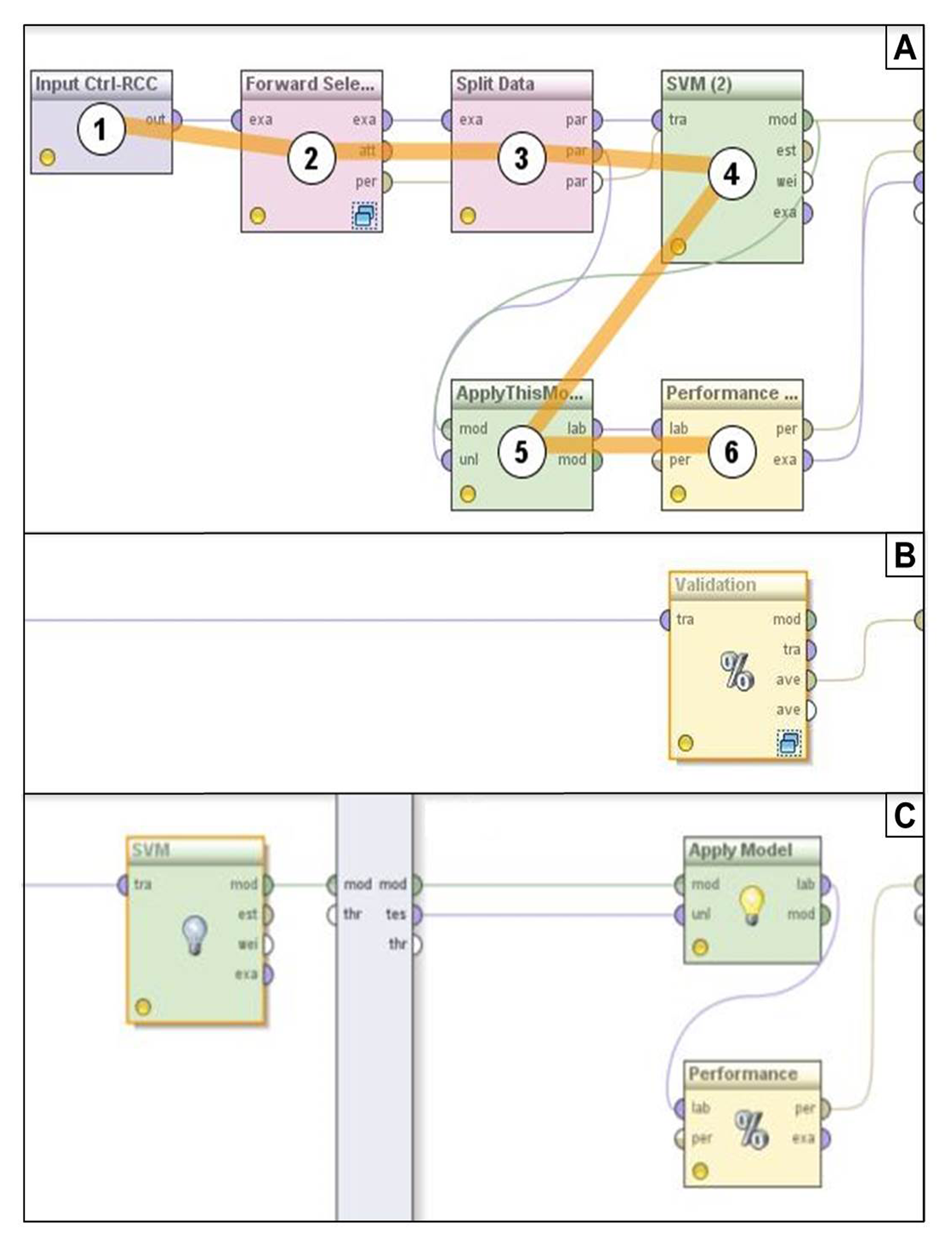

Supplement: Figure S2 — RapidMiner workflow. A: Data is retrieved by the “Input” operator and the feature selection is performed (“Forward Selection operator”). B: Feature selection encapsulates a cross validation process (“Cross Validation operator”) to select the most performing set of features. C: Cross Validation operator encapsulated a k-fold cross validation process. First a classifier is built describing a predetermined set of data classes. Then, the model (a trained SVM) is used for testing new classification examples. The first inner operator (“SVM”) realizes the first step (Training). The second inner operator (“Apply Model”) realizes the second step. Finally, the predictive accuracy of the classifier is estimated by the “Performance” operator (Testing). Blocks 4 and 5 in panel A are given to provide ROC curve analysis (e.g. see Figure S3 for malignant vs benign plus controls and Figure S6 for controls vs ccRCC). (TIF) [file pone.0106684.s002.tif]

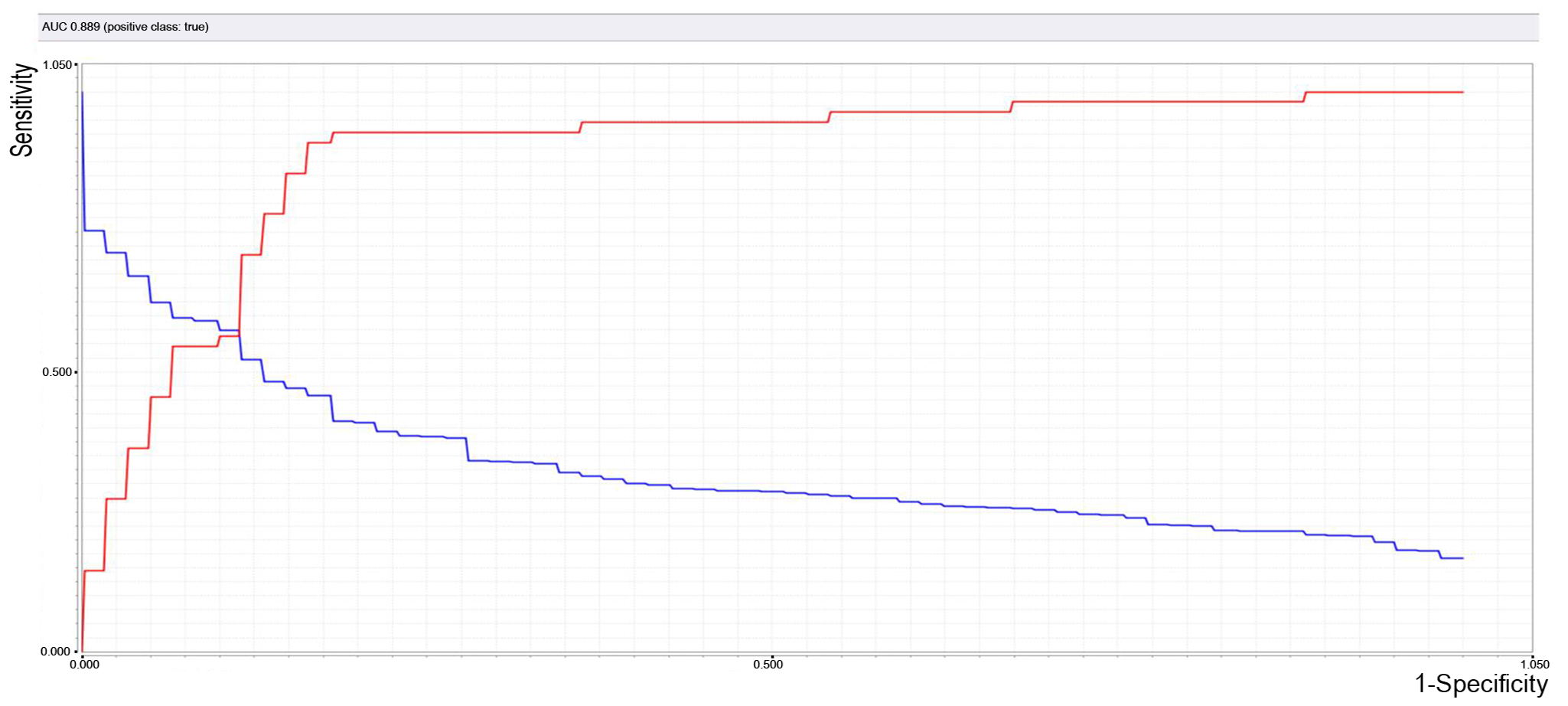

Supplement: Figure S3 — ROC curve analysis of the model discrimination performance when applied to an independent cohort of subjects (malignant vs benign/ctrl). ROC curves are calculated by first ordering the classified examples by confidence. Afterwards all the examples are taken into account with decreasing confidence to plot the false positive rate on the x-axis and the true positive rate on the y-axis. The threshold (blue line) refers to the confidence value of the prediction, i.e. if the confidence of the example to be positive is greater than the threshold, the example will be classified as positive, if the confidence is below the threshold, it will be classified as negative. (TIF) [file pone.0106684.s003.tif]

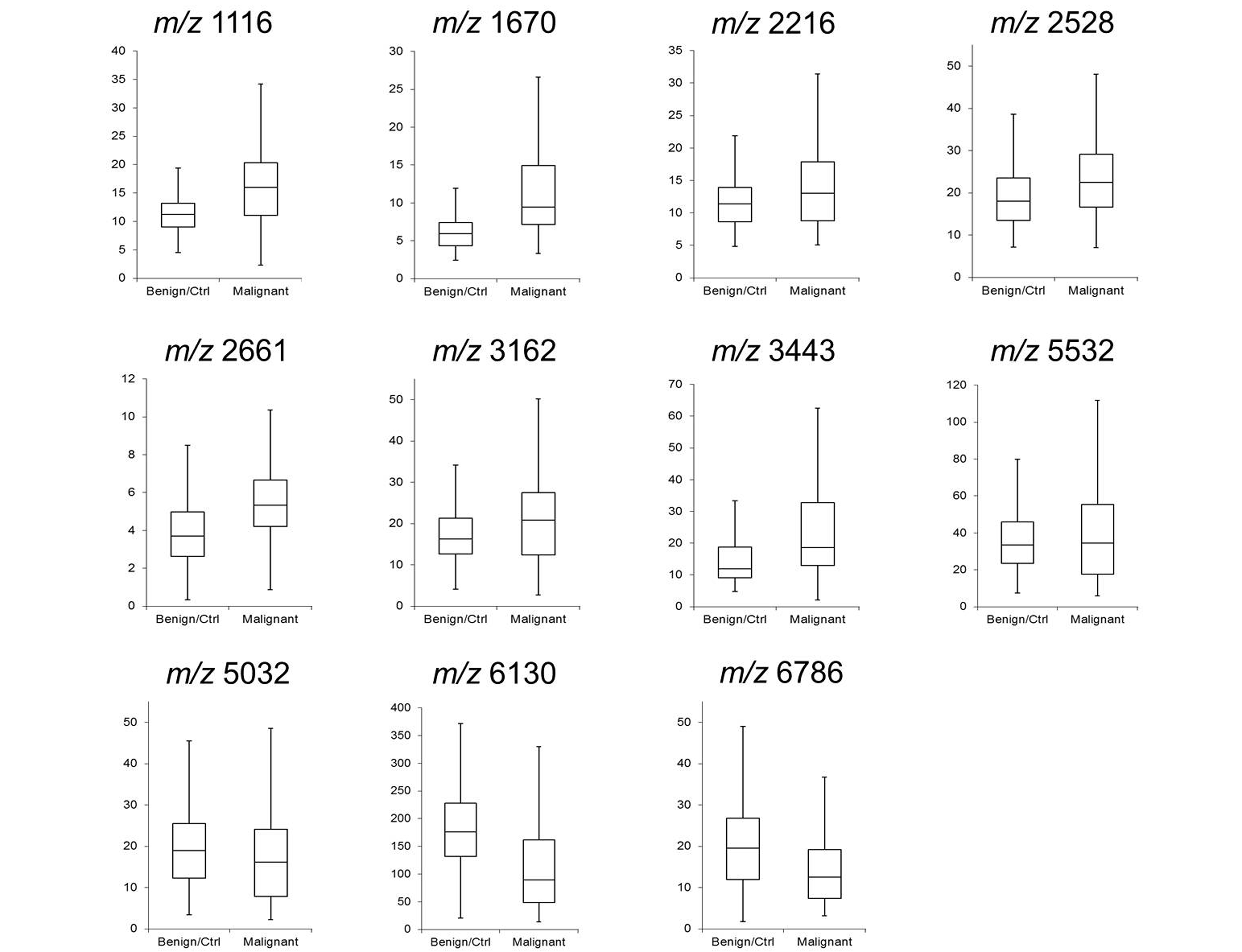

Supplement: Figure S4 — Box-plot of the eleven ions included in the model able to distinguish benign or controls from malignant tumours and statistically different (p<0.05) in the two groups (see Table 3 ). Y-axis refers to arbitrary intensity. (TIF) [file pone.0106684.s004.tif]

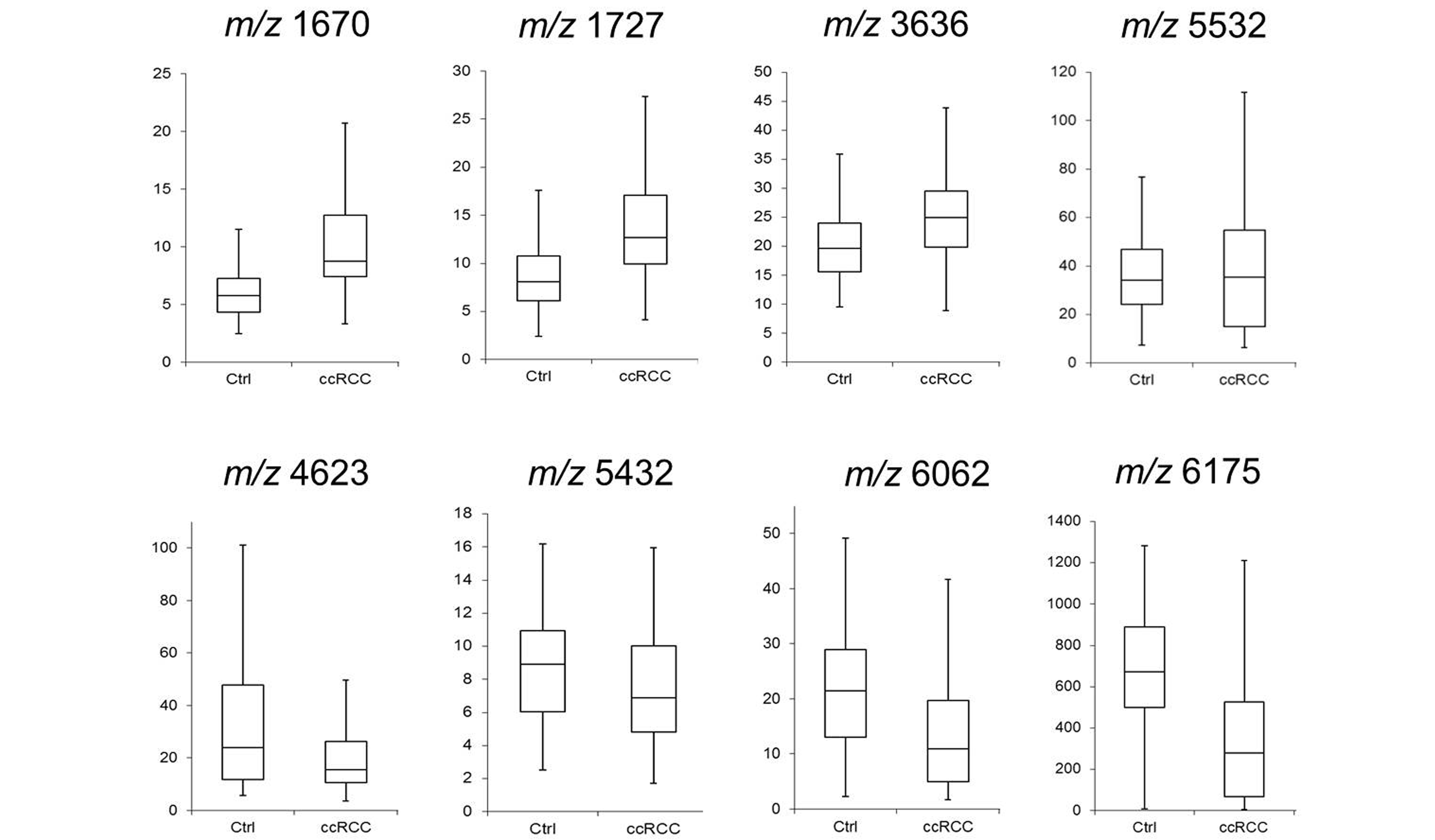

Supplement: Figure S5 — Box-plot of the eight ions included in the model able to distinguish controls from ccRCC and statistically different (p<0.05) in the two groups (see Table 5 ). Y-axis refers to arbitrary intensity. (TIF) [file pone.0106684.s005.tif]

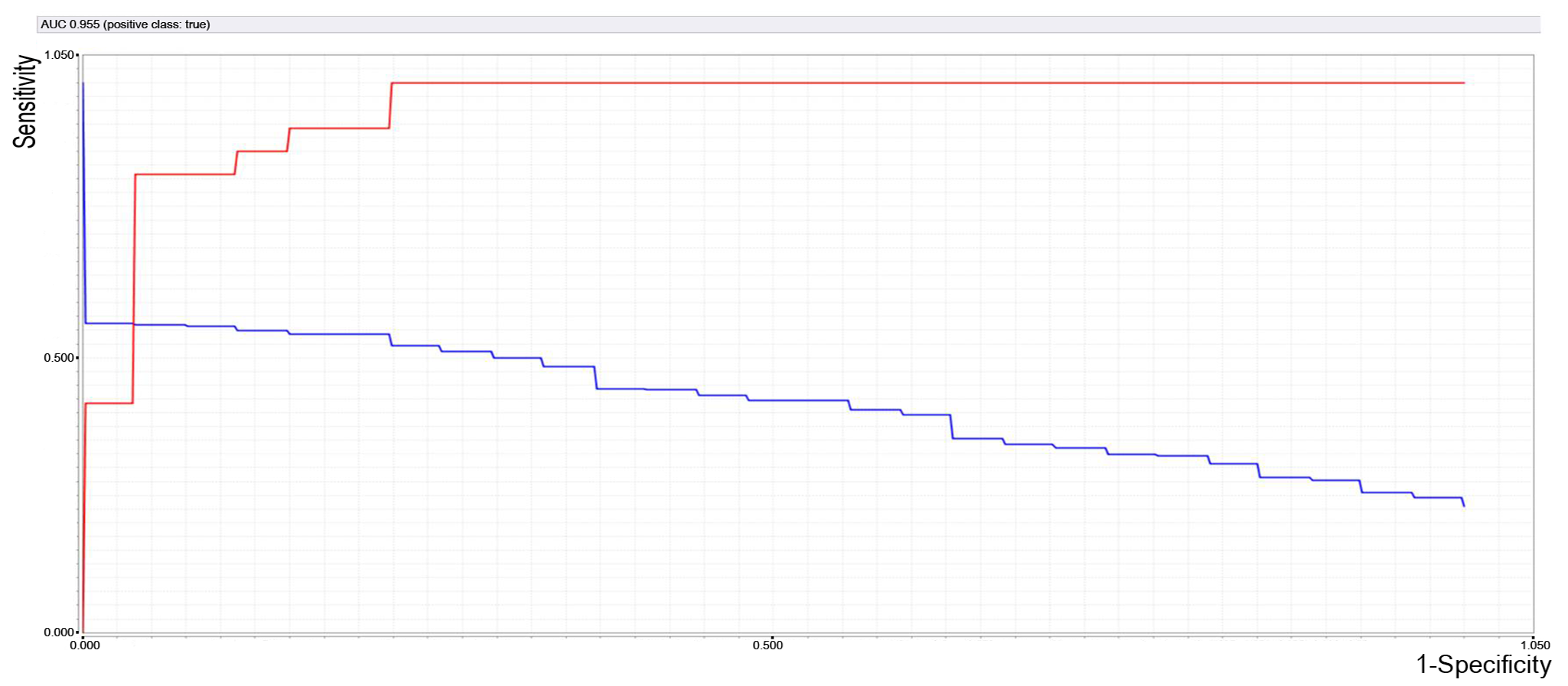

Supplement: Figure S6 — ROC curve analysis of the model discrimination performance when applied to an independent cohort of subjects (controls vs ccRCC). The threshold (blue line) refers to the confidence value of the prediction, i.e. if the confidence of the example to be positive is greater than the threshold, the example will be classified as positive, if the confidence is below the threshold, it will be classified as negative. (TIF) [file pone.0106684.s006.tif]
