# Supplementary material for: Urinary Signatures of Renal Cell Carcinoma Investigated by Peptidomic Approaches
Source: PLoS One. 2014 Sep 9;9(9):e106684. doi: 10.1371/journal.pone.0106684 (PMC4159280; doi:10.1371/journal.pone.0106684)
Supplement: Table S1 — Mean area values of the signals included in the two clusters discriminating malignant tumours from benign renal masses plus controls (A) and ccRCC from controls (B) calculated from raw data before and after spectra elaboration. (DOCX) [file pone.0106684.s007.docx]

| **m/z** | **Raw MS data** | | **MS data after spectra processing** | | **A** |
| --- | --- | --- | --- | --- | --- |
|  |  |  |  |  |  |
|  | **Benign+Ctrl** | **Malignant** | **Benign+Ctrl** | **Malignant** |  |
| 1116 | 10.50 | 22.85 | 12.72 | 30.30 |  |
| 1670 | 8.45 | 13.09 | 6.95 | 12.54 |  |
| 2216 | 14.70 | 17.28 | 12.01 | 15.48 |  |
| 2528 | 16.96 | 22.82 | 19.60 | 25.77 |  |
| 2661 | 6.94 | 12.12 | 4.25 | 11.02 |  |
| 3162 | 15.01 | 19.43 | 17.81 | 23.04 |  |
| 3443 | 35.04 | 46.54 | 27.08 | 42.60 |  |
| 5032 | 13.92 | 12.88 | 19.79 | 17.74 |  |
| 5532 | 41.76 | 44.38 | 35.27 | 42.09 |  |
| 6130 | 172.50 | 103.57 | 176.54 | 109.26 |  |
| 6786 | 19.61 | 14.76 | 24.57 | 15.86 |  |
| 10654 | 2.88 | 2.89 | 2.09 | 2.39 |  |

| **m/z** | **Raw MS data** | | **MS data after spectra processing** | | **B** |
| --- | --- | --- | --- | --- | --- |
|  |  |  |  |  |  |
|  | **Ctrl** | **ccRCC** | **Ctrl** | **ccRCC** |  |
| 1670 | 7.81 | 13.42 | 6.23 | 12.89 |  |
| 1727 | 6.50 | 10.41 | 8.78 | 14.94 |  |
| 2192 | 48.10 | 73.68 | 58.66 | 94.04 |  |
| 3005 | 139.08 | 133.97 | 154.08 | 139.64 |  |
| 3252 | 17.49 | 19.50 | 22.27 | 23.71 |  |
| 3636 | 21.04 | 25.13 | 21.20 | 26.79 |  |
| 4623 | 32.64 | 23.77 | 31.75 | 21.01 |  |
| 5432 | 13.75 | 10.87 | 9.35 | 7.49 |  |
| 5532 | 42.41 | 44.92 | 36.35 | 42.89 |  |
| 5964 | 7.93 | 7.25 | 7.17 | 8.12 |  |
| 6062 | 39.92 | 22.99 | 22.44 | 14.80 |  |
| 6175 | 531.76 | 272.11 | 687.84 | 345.31 |  |
