# Supplementary material for: Urinary Signatures of Renal Cell Carcinoma Investigated by Peptidomic Approaches
Source: PLoS One. 2014 Sep 9;9(9):e106684. doi: 10.1371/journal.pone.0106684 (PMC4159280; doi:10.1371/journal.pone.0106684)
Supplement: Materials S1 — Details of the experimental protocols. (DOCX) [file pone.0106684.s008.docx]

*Urine collection and handling procedure*

Urine was centrifuged at 4°C for 10 min at 1000 g to remove cell debris within one hour from the sample taking. Supernatant was immediately transferred into 2 mL tubes and stored at −80 °C until further use. Sample was thawed once for the automated peptide isolation procedure and for manual urine pools pre-fractionation.

Magnetic beads were used to capture peptides/proteins in urine samples. The profiling kit was employed to purify all samples and checked before the use with a standard light microscope (Dialux EB-20, Leitz, Germany) in order to evaluate dispersion and potential aggregation within the suspension.

The automated extraction procedure was achieved using an 8-channel ClinProt pipetting robot (Bruker Daltonics, Germany) for a greater sample throughput. In particular for C8-MB, 5 µL of beads were used for the analysis of 40 µL of urine sample and mixed with 80 µL of a binding buffer. After the removal of supernatant, the beads were washed twice with 45 µL and once with 30uL of a recommended washing solution. The eluates were obtained by adding 10uL of elution solution (50% acetonitrile in water).

*MALDI-TOF peptide profiling*

In order to obtain MALDI-TOF urinary profiles for all studied patients, the robot automatically performed a MALDI spotting procedure. To this end, 4 μL of the C8 eluate peptide fractions were mixed with 15 μL CHCA matrix solution (0.3 g/L in ethanol/acetone 2:1). Then, 0.8 μL of this mixture was spotted in tetraplicate directly onto a MALDI AnchorChip™ 600/384 target plate (Bruker Daltonics, Germany). The target plates were air-dried and immediately transferred into the MALDI-TOF/TOF mass spectrometer.

Eluates obtained from urine pools MB pre-fractionation were concentrated and desalted following the standard protocol provided by Millipore. An aliquot of each eluate was used for the MALDI-TOF measurement, while the remaining volume for the following nano-LC-ESI-MS/MS analysis. Concerning the MALDI-TOF profiling analysis, 2 μL of eluate were mixed with 4 μL CHCA matrix solution (6.2 g/L CHCA in methanol/acetonitrile/water 50:40:10). Then, 0.8 μL of this mixture were spotted directly onto a MALDI-TOF MTP 384 target plate ground steel F (Bruker Daltonics, Germany) that was afterwards transferred into the MALDI-TOF/TOF mass spectrometer.

*Profile processing*

Spectra processing was performed with FlexAnalysis^TM^ software v. 3.3 (Bruker Daltonics, Germany). Baseline subtraction of all spectra was performed using the “TopHat” algorithm. Then the realignment of spectra was achieved using a subset of peaks common to most, if not all sample profiles. For linear profile data, seven frequent common peaks (at *m/z*-values 1162, 1511, 1681, 1895, 1912, 2236, and 3373) were chosen to create a calibration mass control list with a peak tolerance of 1000 ppm.

An internal calibration was also performed on reflector data using four peaks (at *m/z*-values 1680.93, 1912.06, 2040.17 and 2659.32) with a tolerance in peak assignment of 100 ppm, except for the last one that was 200 ppm. A quadratic calibration algorithm was used for the alignment. Peaks were detected using the SNAP peak detection algorithm with a signal-to-noise (S/N) threshold of 3.

*Peak list selection*

ClinProTools^TM^ software v. 2.2 (Bruker Daltonics, Germany) was used for multiple spectra comparison. Before the peak picking procedure, spectra were normalized against their total ion current and aligned using prominent *m/z* peak values. In order to choose only high quality spectra for the next statistical analysis, data selection was performed based on these parameters: resolution value of 800, “Convex Hull” baseline correction with a baseline flatness of 0.80, null or not recalibratable spectra exclusion. The mean spectrum obtained from each subject data set was used for the statistical elaboration.

Peak finding was carried out with a S/N threshold of 3 and peak areas were calculated using zero level integration type on the total average spectrum. Thereby the software generated a list of peaks (m/z) with their area (as a measure of compound abundance) used for the statistical analysis.

*Peptide identification by nLC-ESI-MS/MS*

Endogenous peptides in the enriched fractions obtained by MB purification of urine pools from controls (n=80) and ccRCC patients (n=80) were also profiled by nLC-ESI-MS/MS. Briefly fractionated samples were first concentrated, then desalted and residual beads removed using Ziptip^TM^ μ-C18 Pipette Tips as already reported [[1](#_ENREF_1)]. Desalted fractions were injected with a draw speed of 1.8 μl/min into a Dionex UltiMate 3000 rapid separation (RS) LC nano system (Thermo Scientific, Germany) coupled online with an Impact HD^TM^ mass spectrometer (Bruker Daltonics, Germany).

Peptide samples were loaded onto a µ-precolumn (Dionex, Acclaim PepMap 100 C18, cartridge, 300 µm i.d. × 5 mm, 5 µm), followed by separation on the analytical 50cm nano column (Dionex, 0.075 mm ID, Acclaim PepMap100, C18, 2 µm), heated to 40°C at a flow rate of 300 nl/min. Multistep 360 min gradients with a ramp from 4 to 35% in 245 min of mobile phase B (0.1% FA/80% CHCN) were used. The column was connected to a CaptiveSpray^TM^ (Bruker Daltonics) equipped with an additional supply of acetonitrile vapor in nitrogen as an ionization enhancer especially for low sample amounts (nanoBooster^TM^, Bruker Daltonics). The mass spectrometer was operated in the data-dependent-acquisition mode to automatically switch between full scan MS and MS/MS acquisition, using the following tune parameters: Funnel 1 RF=400Vpp, Funnel 2 RF=600Vpp, Hexapole RF=400Vpp, pre-puls=10µs. Collision energy was set to 7eV selecting a stepping ramp from 100% to 120% for MS/MS only to facilitate the fragmentation of larger endogenous peptides and N_2_ was used as collision gas. The number of precursor ions was automatically adjusted to fit into a fixed cycle time of 5 sec, to keep the sampling rate constant over the chromatographic peak. The IDAS (Intensity Dependent Acquisition Speed) and RT2 (RealTime Re-Think) functionalities were applied in an MS/MS acquisition window of 300-1221 and 1125-2000 m/z.

Raw MS/MS data were lock-mass corrected (at m/z 1221.9906), deconvoluted and converted to XML peaklists via Compass DataAnalysis^TM^ Software v. 4.1 (Bruker Daltonics Germany). XML data were processed using an in-house Mascot search engine (v2.4.1) through the Mascot Demon tool v 2.4.0. Database searching was restricted to human Swiss-Prot (accessed Apr 2014, 544,996 sequences; 193,815,432 residues). No enzyme and any fixed modification was set in search parameters. Mass tolerances for all identifications were generally set at 20-5 ppm MS and 0.5-0.05 Da MS/MS. Acetyl (N-term) was set as variable modification in Mascot search parameters. Mascot threshold scores for homology and identity and decoy database were used as peptide level filters of peptide significance (False Discovery Rate <1%).

*Expression profile analysis*

Profiles analysis was conducted following the sequence of processes as reported in Figure S1. To apply the correct statistical procedure we first evaluated the assumption of normality and the homogeneity of variance, i.e. we verified whether i) each case/control signal was provided from a normal distribution, and ii) for each signal, both case and control data were provided with the same equal variance (see **Evaluation of parametric assumptions**, **Figure S1, block 1**). Shapiro-Wilk’s test was applied (significance level at 0.05) for testing normality. Barlett and Leven’s test were applied (significance level at 0.05) for testing homogeneity (respectively for normal and non-normal data). The inter-quantile range (IQR) was used to detect and identify outliers. Data instances outside this range were assumed to be “suspiciously extreme” (i.e. outlier) and all the subsequent procedures were applied both by considering and removing this data (see **Outlier detection, Figure S1, block 2)**. As most of our data deviates significantly from the parametric assumptions, the Spearman rank-order correlation (Spearman’rho) was used (see **Correlation analysis, Figure S1, block 3-4)**. In this case, for each signal we obtained, both the strength of association between subject age and signal area (i.e. Spearman’rho) and the statistical significance of this coefficient, i.e. p-value < 0.05 for the null hypothesis, stated no association between the two variables. The following tests were applied to detect differentially expressed signals between control and case subjects: Equal Variance t-test for Normal Data with Equal Variances, Unequal Variance (Welch) t-test for Normal Data with Unequal Variances, Mann-Whitney U-test (Wilcoxon) for Non-normal Data with Equal Variances and Kolmogorov-Smirnov test for Non-normal Data with Unequal Variances (see **Differentially expressed peaks, Figure S1, block 5)** All tests were applied using 0.05 as the significance level.

In order to investigate the diagnostic capabilities of the selected peaks and provide a subset of relevant signals able to discriminate patients (i.e. case) from subjects (controls), we designed a Rapid Miner (RaM) workflow (WF) (see **RapidMiner, Figure S1, block 6**). RaM is a software environment [[2](#_ENREF_2),[3](#_ENREF_3)] for rapid prototyping of machine learning and knowledge discovery (KD) processes. It is currently used for classification, clustering and also data integration tasks. RaM is modelled by a complex nested chain of objects called operators. These operators implement several KD processes, like data pre-processing, performance evaluation, learning algorithms, etc. The user is supported with graphical interfaces, where operators can be dropped as nodes onto the working pane and the data-flow is specified by connecting the operator nodes. In other words, RaM workflows (RaM-WF) represent the sequence of operational process used for specific data mining experiments.

Figure S2 shows the RaM-WF designed for the evaluation of our feature selection and classification problem. Basically, it implements standard Support Vector Machine (SVM) algorithms [[4-6](#_ENREF_4)] to forecast the classification capability of a set of signals. The SVM classifier uses the feature’s intensities (peptides or metabolites) as coordinates in an N-dimensional space (here N=12 see below). It then builds an N-1 dimension by performing a quadratic programming optimization. When the points to be classified are not linearly separable, SVM can use the so-called Kernel-trick (i.e. dot or anova) to transform the sample points to a higher dimensional space. The main issues of this WF are characterized by the *feature selection* (FS) and the *cross-validation* (CV) processes.

**FS** performs a *wrapper* feature (i.e. signal) selection process [[6-8](#_ENREF_6)]. This way, the search in the space of feature subsets is performed by incorporating the classification algorithm within the process. In other words, *wrappers* utilize the classifier as a “black box” to score the subsets of features based on their predictive power. As the number of all feature combinations is exponential in the number of the considered features, the search for the subset which provides the most accurate classification accuracy is often critical for its practical acceptance. To overcome this problem many heuristic methods are used to guide the search of “sub-optimal” sub sets. In our case, we applied a *forward selection* scheme

**CV** encapsulates a k-fold cross validation process (k=10). Cross validation is a two-step process: in the first step, a classifier is built describing a predetermined set of data classes. In the second step, the model (a trained SVM) is used for testing new classification examples; the generalization performance of the classifier is estimated using a new test set. The input data set S is split (split ratio 0.6) into subsets {S_1_, S_2_, ... , S_k_}. The first inner operator (SVM) realizes the learning step described above. SVM is applied k times using at each iteration *i* the set S_i_ as the test set and S\S_i_ as the training set. The second inner operator (model applier) realizes the second step described above. The predictive accuracy (and the other performance measures) of the classifier are then estimated by the performance operator.

The performances of our inference process *a*re given through indices which are broadly applied to measure the *classification performance* of an inference system; *i.e.*, sensitivity, specificity, positive (PPV) and negative predictive values (NPV) [[9](#_ENREF_9)]. The receiver operating characteristic curve analysis (ROC) and area under the curve (AUC) were also evaluated as previously described [[10](#_ENREF_10)].

REFERENCES

1. Gianazza E, Chinello C, Mainini V, Cazzaniga M, Squeo V, et al. (2012) Alterations of the serum peptidome in renal cell carcinoma discriminating benign and malignant kidney tumors. J Proteomics 76 Spec No.: 125-140.

2. Mierswa I, Wurst, M., Klinkenberg, R., Scholz, M., Euler, T. Yale: Rapid prototyping for complex data mining tasks; 2006. pp. 935–940.

3. Zoppis I, Gianazza E, Borsani M, Chinello C, Mainini V, et al. (2011) Mutual Information Optimization for Mass Spectra Data Alignment. IEEE/ACM Trans Comput Biol Bioinform.

4. Cristianini NS-T, J. (2000) An introduction to support vector machines and other kernel-based learning methods: Cambridge University Press.

5. Scholkopf B, Smola A.J. (2001) Learning with Kernels: Support Vector Machines, Regularization, Optimization, and Beyond. Cambridge, MA, USA: MIT Press.

6. Cava C, Zoppis, I., Gariboldi, M., Castiglioni, I., Mauri, G., Antoniotti, M. Copy–Number Alterations for Tumor Progression Inference; 2013; Murcia, Spain. Springer. pp. 104-109.

7. Blum A, Langley, P. (1997) Selection of relevant features and examples in machine learning. Art Intell 97: 245-271.

8. Guyon I, Elissee, A. (2003) An introduction to variable and feature selection. J Machine learning Res 3: 1157-1182.

9. Saeys Y, Inza I, Larranaga P (2007) A review of feature selection techniques in bioinformatics. Bioinformatics 23: 2507-2517.

10. Chinello C, Gianazza E, Zoppis I, Mainini V, Galbusera C, et al. (2010) Serum biomarkers of renal cell carcinoma assessed using a protein profiling approach based on ClinProt technique. Urology 75: 842-847
